# Is Empowerment of Female Radiologists Still Needed? Findings of a Systematic Review

**DOI:** 10.3390/ijerph18041542

**Published:** 2021-02-05

**Authors:** Giulia Fichera, Isolde Martina Busch, Michela Rimondini, Raffaella Motta, Chiara Giraudo

**Affiliations:** 1 Department of Medicine–DIMED, Institute of Radiology, University of Padova, 35122 Padova, Italy; gfichera90@gmail.com (G.F.); raffaella.motta@unipd.it (R.M.); 2 Section of Clinical Psychology, Department of Neuroscience, Biomedicine and Movement Sciences, University of Verona, 37134 Verona, Italy; isoldemartina.busch@univr.it (I.M.B.); michela.rimondini@univr.it (M.R.)

**Keywords:** gender inequality, radiology, female empowerment, women

## Abstract

Considering that radiology is still a male-dominated specialty in which men make up more than two thirds of the workforce, this systematic review aimed to provide a comprehensive overview of the current role of women in radiological imaging, focusing on the main aspects such as career progression, leadership, academic practice, and perceived discrimination. Three electronic databases were searched up to 21 October 2020. To identify additional records, weekly automatic email alerts were set up on PubMed until December 2020 and reference lists of key studies and included papers were screened. Two reviewers independently performed the search, study selection, quality appraisal, data extraction, and formal narrative synthesis. In case of disagreement, a third reviewer was involved. Across the 61 included articles, women worked more often part-time and held fewer positions of power in hospitals, on editorial boards, and at the academic level (associate and full professors). Women were less often in relevant positions in scientific articles, had fewer publications, and had a lower H-index. Discrimination and sexual harassment were experienced by up to 40% and 47% of female radiologists, respectively. Our study highlights that women in radiology are still underrepresented and play a marginal role in the field, struggling to reach top and leading positions.

## 1. Introduction

As stated by Paik in the Journal of the American Medical Association [1], “A generation ago a woman’s role in medicine was that of a patient”. Fortunately, we are witnessing major advancements. Nowadays, the number of women pursuing a medical career is constantly increasing [2] because, in the last decades, strong efforts have been made to enhance gender equality in medicine and, overall, in science. For instance, “to achieve full and equal access to and participation in science for women and girls”, the United Nations General Assembly declared 11 February as International Day of Women and Girls in Science [3]. Contemporary feminist movements such as the Women’s March as well as foundations such as the Global Fund for Women demonstrate that we are living in a time where women are starting to become empowered [4,5].

Nevertheless, male predominance is still widespread at various levels of medical training [6,7] and women in the academic environment occupying leading positions are underrepresented [6].

Regarding, in particular, women in radiology and related sciences, more than a century has passed since Elizabeth Fleischman opened her own X-ray laboratory in San Francisco in 1896, Florence Ada Stoney became the first female radiologist in the UK in 1898, and Marie Curie was officially acknowledged with the Nobel Prize in Physics in 1903. Regarding this latter achievement, it should not be omitted that the Nobel Prize Committee only recognized the equal contribution of Marie Curie because Pierre Curie refused to accept the award unless the crucial role of his wife was acknowledged [8]. More recently, initiatives such as the American Association for Women in Radiology (AAWR), promoting opportunities and networking, represent a crucial support for women in clinical, research, and leadership positions [9]. Nevertheless, numerous challenges still lie ahead and ample opportunities for improvement remain.

In fact, to date, according to the American Medical Association, radiology is still a male-dominated specialty in which men make up 73.2% of the resident workforce [10]. Several potential reasons for this gender disparity have been suggested, including the fear of radiation exposure, the central role of technology, and the limited interaction with patients [11,12].

Given that healthcare systems closing the gender gap have shown to better perform in terms of organization and clinical outcomes, an increase in the number of women practicing radiology is urgently needed and women and men should also be equally treated and given the same opportunities in this medical field [2,12,13,14,15].

Aiming to provide a comprehensive overview of the current role of women in radiological imaging, we performed a systematic review of the literature addressing gender inequality in radiology.

## 2. Materials and Methods

### 2.1. Search Strategy

The following search strategy was used to perform a systematic search of three electronic databases (i.e., PubMed, Scopus, and Web of Science) up to 21 October 2020: ((gender OR women) AND (inequality) AND (radiology)). No restrictions on the time interval or language were applied. Supplementary Text S1 provides a detailed record of the applied search strategy for each electronic database.

Duplicates were automatically removed using Mendeley (Mendeley Software, 2009–2013 Mendeley Ltd., London, UK). A weekly automatic email alert was set on PubMed to receive notifications of newly published literature (22 October 2020–14 December 2020).

The following inclusion criteria were applied:

- Original articles, case studies, and special reports;
- Articles written in English, German, French, or Italian (languages spoken by the authors);
- Studies reporting quantitative and/or qualitative data on the role of women in radiology;
- Articles covering at least one of the following domains: gender distribution in the field of radiology, also including radiological societies, academia, residency programs, leadership positions; part-time and full-time employment; income; job satisfaction; perceived discrimination; sexual harassment; academic aspects (authorship, H-index, editorial board membership, grant support); mentorship for residents; private life (relationship, child rearing).

The following exclusion criteria were used:

- Literature reviews of any type, general discussion and opinion papers, comments, editorials, case reports, and book chapters;
- Non-English, Non-German, Non-French, or Non-Italian articles;
- Articles not covering any of the domains mentioned above.

Two reviewers (G.F. and I.M.B.) with 4 years of experience in diagnostic imaging and 7 years of experience in psychology and public health, respectively, independently screened titles and abstracts of the records. Full texts considered as potentially eligible by at least one of the two reviewers were then independently evaluated; in case of dissent about the inclusion of particular full texts, a third reviewer (M.R., with 20 years of experience in psychology and public health) was involved to make the final decision. To identify additional records, the reference lists of key studies and the included papers were screened.

### 2.2. Quality Appraisal

The methodological quality of the included articles was independently assessed by two reviewers (I.M.B. and R.M.) using the Mixed Methods Appraisal Tool (MMAT) (Version 2018) [16], which allows rating qualitative, quantitative, and mixed methods studies, based on a 5-item criteria list. In case of disagreement, a third reviewer (M.R.) was involved.

### 2.3. Data Extraction and Analysis

Study characteristics such as publication year, journal, country, and study design of each included paper were extracted. For each included paper, we assessed the overall number of female authors/co-authors, also recording if any were in a relevant position (i.e., first, second, last, and/or corresponding author). We recorded publications’ impact indexes (i.e., current quartile and impact factor) of the journals where the studies were published.

The main results of the included studies were extracted and summarized in a formal narrative synthesis organized around five main thematic areas:

1. Women in radiology;
2. Work environment;
3. Academic practice;
4. Training and mentorship;
5. Private life.

Descriptive statistics of the collected variables, also grouped according to the homogeneity of data of the selected studies, were performed using SPSS (IBM SPSS Statistics version 26, IBM Armonk, NY, USA).

## 3. Results

**Figure 1:**
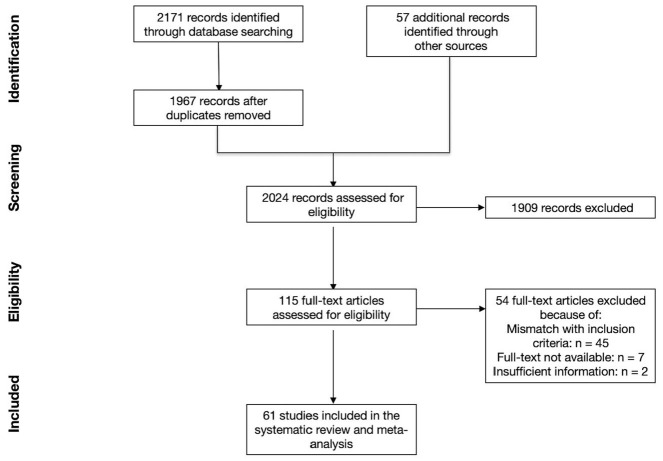
PRISMA flow diagram.

Our search provided an overall amount of 2024 records. After screening for titles and abstracts, we assessed 115 full texts for eligibility. Fifty-four articles [17,18,19,20,21,22,23,24,25,26,27,28,29,30,31,32,33,34,35,36,37,38,39,40,41,42,43,44,45,46,47,48,49,50,51,52,53,54,55,56,57,58,59,60,61,62,63,64,65,66,67,68,69,70] were then excluded because of different reasons such as a mismatch with inclusion criteria and unavailability of the full text (see Figure 1 and Supplementary Table S1). A total number of 61 articles were included [6,7,11,12,71,72,73,74,75,76,77,78,79,80,81,82,83,84,85,86,87,88,89,90,91,92,93,94,95,96,97,98,99,100,101,102,103,104,105,106,107,108,109,110,111,112,113,114,115,116,117,118,119,120,121,122,123,124,125,126,127].

Fifty-two articles (85.2%) fulfilled at least four of the quality appraisal criteria [7,11,12,71,72,75,76,78,79,80,81,82,84,86,87,88,89,91,92,93,94,95,96,97,99,100,101,102,103,104,105,106,107,108,109,110,111,112,113,114,115,116,117,118,119,121,122,123,124,125,126,127] (see Supplementary Table S2).

### 3.1. Characteristics of the Included Studies

The main characteristics of the primary studies are summarized in Table 1.

**Figure 2:**
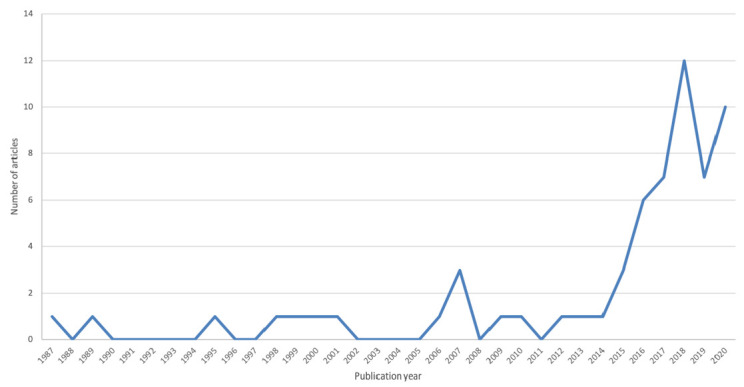
Publishing timeline of the included studies.

All included articles were written in English and published between 1989 and 2020 [6,7,11,12,71,72,73,74,75,76,77,78,79,80,81,82,83,84,85,86,87,88,89,90,91,92,93,94,95,96,97,98,99,100,101,102,103,104,105,106,107,108,109,110,111,112,113,114,115,116,117,118,119,120,121,122,123,124,125,126,127] (Figure 2). Most of the studies were conducted in the United States of America (USA) (*n* = 35) [7,11,73,74,77,79,80,81,82,83,84,85,87,88,90,91,92,94,95,97,100,102,103,105,106,107,108,113,114,115,116,119,122,123,124] alone or in collaboration with other countries (*n* = 12) [6,71,75,93,96,104,110,111,117,121,127]. Five articles were published in Canada [86,98,99,111,126] and two in France [76,109]. One article was an international multicenter study [118], and two resulted from a collaboration between Australia and Ireland [89] and Vietnam and Malaysia [12], respectively. One article each was published in Italy [101], Switzerland [78], Saudi Arabia [72], the United Kingdom [120], and South Korea [125].

Most of the articles appeared in Q1 journals (80.3%), with an impact factor ranging from 0 [12,72,98,100,104,118,127] to 7.931 [80,81,94,97]. The three most common journals were the American Journal of Roentgenology (i.e., 15 articles) [71,83,84,86,87,88,90,95,96,105,107,111,114,121,125], the Journal of the American College of Radiology (i.e., 12 articles) [7,73,74,77,79,82,99,102,106,113,115,126], and Academic Radiology (i.e., nine articles) [11,91,92,93,103,108,116,117,119].

A woman was the first author in 45 (73.8%) articles [6,11,73,76,78,80,81,82,83,84,86,87,88,89,90,92,93,94,95,96,97,98,99,102,103,104,105,106,107,108,109,110,111,115,116,118,119,120,121,122,123,125,126,127], the second in 27 (44.3%) [6,7,11,72,74,77,78,79,80,81,82,85,88,91,92,97,98,99,103,113,114,115,117,118,119,121,126], and the last in 22 (36.1%) [77,78,84,85,87,88,90,91,92,93,98,99,102,105,106,118,119,120,121,122,123,124,125,126]. In five out of the 61 included articles (8.2%), there were no women in a relevant position [12,71,75,100,101] (Table 1).

### 3.2. Women in Radiology

The average number of women in radiology, including societies’ members and subspecialists (e.g., interventional and abdominal radiologists), ranged from 8.2% to 35.95% (vs. a range for men from 64.05% to 90%) [6,11,12,75,77,80,83,85,94,95,98,100,103,105,111,112,113,114,116,121,127].

In the selected studies, the percentage of part-time employment ranged from 11% to 50% for women and from 1% to 22% for men [77,78,81,102,105,119,120]. Several studies found similar working hours for both genders (~50 h/week) [81,82,84,105,119]. Nevertheless, in the survey of Vydareny and colleagues, male associate professors reported working more hours than their female counterparts [119], and Lewis and colleagues, considering all types of practices, showed that men work significantly more hours than women [97].

In terms of income, the survey by Chertoff et al. reported that women earn less than men in both full-time and part-time employment (i.e., USD 229,884 ± 4470 vs. USD 269,301 ± 4310 and USD 134,161 ± 5290 vs. USD 160,193 ± 19,220, respectively) [81]. On the contrary, Kapoor et al., screening salary information of state-employed academic radiologists, found similar salaries for both genders (USD 290,660 for men vs. USD 289,797 for women) [95].

Men were overrepresented in leadership positions in most of the studies (percentage range: men 68.09–91.8% vs. women 8.2–31.9%) [6,11,75,77,100,104,111,112,117,127], aside from one on breast imaging which showed a countertendency (59.7% of women vs. 40.3% of men) [96].

### 3.3. Work Environment

While three studies found similar levels of job satisfaction between men and women [82,84,97], two studies reported significantly higher satisfaction among men [78,101].

Perceived discrimination was assessed in two studies. Namely, Deitch [84] reported that 40% of women and 1% of men had experienced discrimination at work. Along the same line, 31% of female residents and fellows surveyed by Pyatrigorskaya et al. [110] felt discriminated against during application processes for positions or conferences.

Sexual harassment and unwanted sexual attention were addressed in four studies [84,87,90,110]. Frank et al. [90] reported that 45% of female radiologists experienced sexual harassment and Pyatigorskaya et al. [110] noted its occurrence for 10% of French female residents and fellows. Englander and colleagues [87] highlighted that it was more frequent during practice than training (47% vs. 22%), while, previously, Deitch et al. [84] showed that unwanted sexual attention was higher during training than practice (39% vs. 21% on average).

### 3.4. Academic Practice

**Figure 3:**
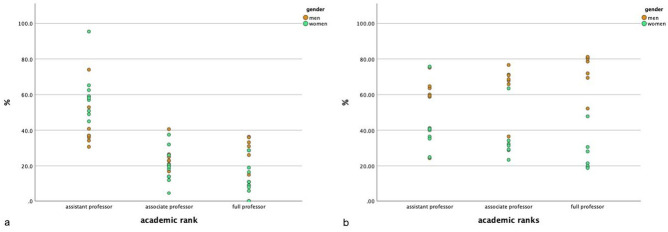
Distribution of academic ranks based on within-gender analyses (**a**) and analyses at the academic level (**b**).

Twenty-four studies calculated the distribution of gender in academic rankings [6,12,75,83,85,94,95,96,97,100,102,104,111,112,117,119,121,123,127]. Due to the heterogeneous ways of computing the gender distribution at each main academic level (i.e., assistant, associate, and full professor), in the following, we separately group the studies which performed within-gender analyses (e.g., numerator: number of women assistant professors; denominator: total amount of women in the sample) from those which assessed the distribution of gender at each academic level (e.g., numerator: number of women assistant professors; denominator: total amount of men and women assistant professors in the sample) (Figure 3a,b, respectively).

As depicted in Figure 3a, most of the women and men were assistant professors (range 45% to 95.4% for the former and 30.7% to 74% for the latter) [75,83,85,94,95,102,119,121,123]. Moreover, in two studies, it occurred that there were no full professors among all women [75,85].

Figure 3b illustrates that a higher percentage of men than women reach associate and full professor positions [6,12,96,97,100,103,111,112,127]. It should be noted that among the studies represented in graph b, four addressed subspecialties (i.e., one study each for abdominal, breast, musculoskeletal radiology, and neuroradiology) and only the study of Khurshid et al. [96], in the field of breast imaging, reported a higher number of female than male associate professors (i.e., 63.5% vs. 36.5%).

Overall, in scientific journals, women were poorly represented as authors (12–28% of women vs. 72–88% of men) [7,99,124]. Similarly, it emerged that women were less often in a relevant position (first author range: female 17–30% vs. male 70–84%; last author range: female 9–25% vs. male 75–91%) [76,79,99,103,107,124]. Nevertheless, two studies observed, along the years, a trend towards an increase in female first, last, and corresponding authorship [109,125].

Two studies reported a higher median number of publications for female assistant and full professors, respectively [111,112], but, on average, women published less than men (range of mean publication number: men 21.99–58.69 vs. women 14.4–35.59) [94,95,123,127] and had a lower H-index (range of mean H-index: 10–14.65 for men vs. 4.29–11.3 for women) [75,83,127].

Among the editorial board members, women were underrepresented (women: ranging from 13.6% to 19.3%; men: ranging from 80.87% to 86.4%) [71,93,108]. Moreover, Piper et al. noted that in four radiology journals (i.e., Radiology, American Journal of Radiology, JACR, and Academic Radiology), since each journal’s inception, a woman never served as chief editor [108].

More men than women reported grant support [119,123] and received a higher number of grants [78,83,94,95].

### 3.5. Training and Mentorship

Men outnumbered women as applicants, residents, and fellows in radiology (range for men: 71.8–77.2%; range for women: 24–28.1%) [74,79,80,92,122]. One study reported a greater number of women only in pediatric fellowships [122]. Further, 19% of women responding to a survey by Englander et al. said that they were discouraged from pursuing a career in radiology.

Mentorship was addressed in several studies [73,81,86,88,89,91,106,115,126]. Three articles noted that women with a mentor were underrepresented (range for women: 0–40.3%; range for men: 23.3–64%) [72,78,118]. One study showed that for female medical students, the influence of role models was one of the three most important factors in choosing a career in radiology [73]. Similarly, in a study by Donovan et al., a greater proportion of female than male program directors noted the major role mentorship had played in their own careers (68% female vs. 35% male) as well as the importance of female mentors for female radiology residents [86].

### 3.6. Private Life

The majority of female radiologists turned out to be married or in a partnership (67–83.3%) [78,87,90]. The percentage of female radiologists with children ranged from 48% to 69.8% [78,87,90]. In one article, 72.9% of women considered child rearing as a reason for working part-time in contrast to 5% of the surveyed men [81]. Child rearing was considered as barrier to career development by female radiologists interviewed by Piltch-Loeb and colleagues and by 85.7% of women and 70.4% of men assessed by Daldrup-Link et al. [82,106]. Similarly, pregnancy-related issues, difficulties in combining work duties with family life, and maternity leave were mentioned by female interventional radiologists as obstacles [120].

## 4. Discussion

The first interesting point emerging from our systematic review is represented by the fact that most of the papers were published in the last five years, suggesting that only recently the radiological community approached this topic. Moreover, the high number of American studies and the paucity of papers from other countries indicate that American radiologists are ahead in openly discussing the matter of gender equality [7,11,73,74,77,79,80,81,82,83,84,85,87,88,90,91,92,94,95,97,100,102,103,105,106,107,108,113,114,115,116,119,122,123,124]. This evidence is also reflected by our finding that most of the articles were published in high-indexed American journals.

Across countries, women in radiology are still underrepresented and hold fewer leadership positions. Breast imaging seems to be an exception with a higher proportion of women not only in the workforce and in executive positions but also as associate professors [96].

Regarding, in particular, academic careers, our results show that women still have lower H-indexes and a lower number of publications and are less frequently in relevant positions in papers. Although this could be seen as a causality dilemma, wondering if women succeed less because they are given less chances or have less chances to succeed because they are not as good as their male counterpart, it can hardly be denied that the difficulties of women to reach top positions are mainly due to unequal opportunities [129], as also recently stated in a Lancet editorial, “the world is still reckoning with pervasive and inexcusable gender inequality underpinned by bias and sexism, and research and health care are not exception” [130].

The “leaky pipeline” is also reflected by the distribution of authorship in our selected studies [131]. Indeed, women were first authors in more than two thirds of the papers but last authors only in around one third. This indicates that even if it comes to the matter of gender inequality, in most of the cases, men are still leading research projects. While this finding may also imply that male radiologists are genuinely interested in the topic of gender differences, it primarily suggests that more men than women hold senior positions in academic radiology, that is, positions which give them the opportunity to supervise those projects.

Similarly, our study showed that the number of women assistant professors is higher than that of men but that an opposite trend for associate and full professorship exists. One might argue that the disparities observed at the more senior level will become less prevalent with time, as the natural progression from assistant professors to professors occurs. However, our finding is based not only on recent studies but also on older articles published in 1987 [123], 1989 [85], and 2000 [119], thus suggesting that in the last two decades, such progression did not naturally occur. Considering, however, that there has been a growing awareness of the issue of gender inequality in radiology and that the percentage of female authorship has been steadily increasing in recent years [99,107,109], we expect these disparities to diminish in the future.

Challenges that women have to face in their practice are not only associated with their career but also with the work environment. For instance, they are still affected by rigid gender roles, as indicated by the finding that larger shares of women work part-time and struggle in aligning work and family, considering child rearing as an obstacle [81,82,106,120]. Moreover, women feel discriminated against and many are still victims of sexual harassment throughout their career [84,87,90]. In creating paths of change, tailored programs promoting respect in the workplace should be implemented internationally [132,133]. Further, to foster inclusive workplaces, family-supportive policies, flexible work opportunities, and programs ensuring a fair distribution of promotions should be encouraged [66]. Despite some controversial results in the assessed literature regarding the gender pay gap [81,95], salary equity should be always guaranteed [66].

Last, medical schools and residency programs certainly play a crucial role in closing the gender gap. Since several studies addressed the importance of mentoring in choosing a career in radiology and one even highlighted the significant impact of role models on women, we call for mentoring programs such as the one at the Massachusetts General Hospital [134] and the Women in Radiology Group at Indiana University [135].

A number of weaknesses have to be considered.

First, intrinsic limitations associated with systematic reviews such as the potential impact of studies not detected by the search process, also because of the relatively short search strategy, must be taken into account. To compensate for this shortcoming, we additionally screened the reference lists of the included studies and key papers.

Moreover, the primary studies differed in the data analysis and description (e.g., median and mean values for the number of women in leadership positions in the academic field) of some variables, hampering their synthesis.

Although we did not perform any in-depth evaluation of different subspecialties due to the small number of articles focused on each subspecialty, we synthesized the main findings. For instance, as mentioned above, breast imaging showed a higher female representation, while, on the contrary, in interventional, abdominal, and musculoskeletal radiology, a male predominance was reported.

## 5. Conclusions

In conclusion, our systematic review highlights that women in radiology are still underrepresented and play a marginal role, struggling to reach top and leading positions. Thus, continued efforts are still needed to see the vision of Sandra Day O’Connor, the first female justice of the Supreme Court of the United States, who realized: “As women achieve power, the barriers will fall. As society sees what women can do, as women see what women can do, there will be more women out there doing things, and we’ll all be better off for it” [136].

## Figures and Tables

**Table 1:** Characteristics of the 61 included studies.

Authors	Year	Country	Journal	Quartile (Q) *,^†^	Impact Factor ^†^	Relevant Authorship Positions Occupied by Women
Abdellatif et al. [71]	2019	Canada/USA	American Journal of Roentgenology	Q1	3.013	/
Abduljabbar et al. [72]	2020	Saudi Arabia	Interactive Journal of Medical Research	/	/	2nd author
Ahmadi et al. [6]	2018	Canada/USA	American Journal of Neuroradiology	Q1	3.381	1st author 2nd author
Arleo et al. [73]	2016	USA	Journal of the American College of Radiology	Q1	4.268	1st author ^‡^
Baker et al. [74]	2006	USA	Journal of the American College of Radiology	Q1	4.268	2nd author ^‡^
Battaglia et al. [75]	2018	Canada/USA	Emergency Radiology	Q3	1.010	/
Bernard et al. [76]	2020	France	European Radiology	Q1	4.101	1st author
Bluth et al. [77]	2015	USA	Journal of the American College of Radiology	Q1	4.268	2nd author last author
Buddeberg-Fischer et al. [78]	2012	Switzerland	European Journal of Radiology	Q1	2.687	1st author 2nd author last author ^‡^
Campbell et al. [79]	2017	USA	Journal of the American College of Radiology	Q1	4.268	2nd author
Campbell et al. [7]	2018	USA	Journal of the American College of Radiology	Q1	4.268	2nd author
Cater et al. [11]	2018	USA	Academic Radiology	Q1	2.488	1st author ^‡^ 2nd author
Chapman et al. [80]	2013	USA	Radiology	Q1	7.931	1st author 2nd author
Chertoff et al. [81]	2001	USA	Radiology	Q1	7.931	1st author ^‡^ 2nd author
Daldrup-Link et al. [82]	2019	USA	Journal of the American College of Radiology	Q1	4.268	1st author ^‡^ 2nd author
Deipolyi et al. [83]	2020	USA	American Journal of Roentgenology	Q1	3.013	1st author ^‡^
Deitch et al. [84]	1998	USA	American Journal of Roentgenology	Q1	3.013	1st author last author
Dial et al. [85]	1989	USA	Academic Medicine	Q1	5.354	2nd author last author
Donovan [86]	2010	Canada	American Journal of Roentgenology	Q1	3.013	1st author ^‡^
Duc et al. [12]	2020	Vietnam/Malaysia	Acta Informatica Medica	Q3	/	/
Englander et al. [87]	2018	USA	American Journal of Roentgenology	Q1	3.013	1st author ^‡^ last author
Fielding et al. [88]	2007	USA	American Journal of Roentgenology	Q1	3.013	1st author ^‡^ 2nd author last author
Foo et al. [89]	2020	Australia/Ireland	Clinical Radiology	Q2	2.118	1st author ^‡^
Frank et al. [90]	1999	USA	American Journal of Roentgenology	Q1	3.013	1st author last author
Grimm et al. [91]	2017	USA	Academic Radiology	Q1	2.488	2nd author last author
Hewett et al. [92]	2016	USA	Academic Radiology	Q1	2.488	1st author ^‡^ 2nd author last author
Joshi et al. [93]	2020	USA/India	Academic Radiology	Q1	2.488	1st author last author ^‡^
Kapoor et al. [94]	2017	USA	Radiology	Q1	7.931	1st author ^‡^
Kapoor et al. [95]	2017	USA	American Journal of Roentgenology	Q1	3.013	1st author ^‡^
Khurshid et al. [96]	2018	Canada/USA	American Journal of Roentgenology	Q1	3.013	1st author
Lewis et al. [97]	2007	USA	Radiology	Q1	7.931	1st author ^‡^ 2nd author
Li et al. [98]	2020	Canada	Current Problems in Diagnostic Radiology	Q3	/	1st author ^‡^ 2nd author last author
Liang et al. [99]	2015	Canada	Journal of the American College of Radiology	Q1	4.268	1st author ^‡^ 2nd author last author
Maddu et al. [100]	2020	USA	Current Problems in Diagnostic Radiology	Q3	/	/
Magnavita [101]	2013	Italy	European Journal of Radiology	Q1	2.687	/
McDonald et al. [102]	2017	USA	Journal of the American College of Radiology	Q1	4.268	1st author ^‡^ last author
O’Connor et al. [103]	2018	USA	Academic Radiology	Q1	2.488	1st author ^‡^ 2nd author
O’Neill et al. [104]	2019	Canada/USA	Current Problems in Diagnostic Radiology	Q3	/	1st author
Owen et al. [105]	1995	USA	American Journal of Roentgenology	Q1	3.013	1st author last author
Piltch-Loeb et al. [106]	2020	USA	Journal of the American College of Radiology	Q1	4.268	1st author last author ^‡^
Piper et al. [107]	2016	USA	American Journal of Roentgenology	Q1	3.013	1st author ^‡^
Piper et al. [108]	2018	USA	Academic Radiology	Q1	2.488	1st author
Pyatigorskaya et al. [109]	2017	France	Diagnostic and Interventional Imaging	Q2	2.527	1st author ^‡^
Pyatigorskaya et al. [110]	2017	USA/France	Diagnostic and Interventional Imaging	Q2	2.527	1st author ^‡^
Qamar et al. [111]	2018	Canada/USA	Skeletal Radiology	Q2	1.618	1st author ^‡^
Qamar et al. [112]	2020	Canada	American Journal of Roentgenology	Q1	3.013	1st author
Rosenkrantz et al. [113]	2018	USA	Journal of the American College of Radiology	Q1	4.268	2nd author
Rosenkrantz et al. [114]	2019	USA	American Journal of Roentgenology	Q1	3.013	2nd author
Roubidoux et al. [115]	2009	USA	Journal of the American College of Radiology	Q1	4.268	1st author ^‡^ 2nd author
Sadigh et al. [116]	2019	USA	Academic Radiology	Q1	2.488	1st author ^‡^
Shah et al. [117]	2007	USA/Brazil	Academic Radiology	Q1	2.488	2nd author
Vernuccio et al. [118]	2019	Italy/France/UK/Spain/Turkey/ Mexico/ Philippines/ India/Egypt/ Argentina/ Mongolia/South Korea/USA	Insights into Imaging	Q1	/	1st author ^‡^ 2nd author last author
Vydareny et al. [119]	2000	USA	Academic Radiology	Q1	2.488	1st author ^‡^ 2nd author last author
Wah et al. [120]	2018	UK	CardioVascular and Interventional Radiology	Q2	2.034	1st author ^‡^ last author
Wang et al. [121]	2019	Canada/USA	American Journal of Roentgenology	Q1	3.013	1st author ^‡^ 2nd author
West et al. [122]	2016	USA	Journal of Racial and Ethnic Health Disparities	Q1	1.661	1st author
Whitley et al. [123]	1987	USA	Investigative Radiology	Q1	5.156	1st author ^‡^ last author
Xiao et al. [124]	2018	USA	Journal of Vascular and Interventional Radiology	Q1	3.037	last author
Yun et al. [125]	2015	South Korea	American Journal of Roentgenology	Q1	3.013	1st author ^‡^ last author
Zener et al. [126]	2016	Canada	Journal of the American College of Radiology	Q1	4.268	1st author ^‡^ 2nd author last author
Zulfiqar et al. [127]	2020	USA/Canada/UK	Current Problems in Diagnostic Radiology	Q3	/	1st author

* according to www.scimagojr.com [128]; ^†^ current; ^‡^ also corresponding author.

## Data Availability

The data presented in this study are available on request from the corresponding author.

## Supplementary Materials

The following are available online at https://www.mdpi.com/1660-4601/18/4/1542/s1, Supplementary Text S1: Search strategy and retrieved records from each electronic databases; Supplementary Table S1: List of excluded studies; Supplementary Table S2: Quality appraisal of included studies.
