# Supplementary material for: Is Empowerment of Female Radiologists Still Needed? Findings of a Systematic Review
Source: Int J Environ Res Public Health. 2021 Feb 5;18(4):1542. doi: 10.3390/ijerph18041542 (PMC7915271; doi:10.3390/ijerph18041542)
Supplement: Supplementary file 1 [file ijerph-18-01542-s001.pdf]

Supplementary Text S1. Search strategies and retrieved records from each electronic database

1. PubMed

Date of search: 21st of October 2020

Search strategy:
#1 (gender OR women)
#2 inequality
#3 radiology

#1 AND #2 AND #3

n findings: 268

2. Scopus

Date of search: 21st of October 2020

Search strategy:
((gender OR women) AND (inequality) AND (radiology))

n findings: 1,898

3. Web of Science Core Collection

Date of search: 21st of October 2020

Search strategy:
#1 TS=(gender OR women)
#2 TS=(inequality)
#3 TS=(radiology)
#4: #1 AND #2 AND #3

n findings: 5

Supplementary Table S1. List of excluded studies

	Study	Reason for exclusion
1.	Abdellatif, W.; Ding, J.; Jalal, S.; Chopra, S.; Butler, J.; Ali, I. T.; Shah, S.; Khosa, F. Leadership Gender Disparity Within Research-Intensive Medical Schools: A Transcontinental Thematic Analysis. *J Contin Educ Health Prof* **2019**, *39*, 243–250.	Mismatch with the inclusion criteria
2.	Aldrich, M.C.; Cust, A.E.; Raynes-Greenow, C. International Network for Epidemiology in Policy. Gender equity in epidemiology: a policy brief. *Ann Epidemiol* **2019**, *35*, 1–3.	Mismatch with the inclusion criteria
3.	Anim-Sampong, S.; Arthur, L.; Nkansah, J. A.; Botwe, B. O. Women in Radiography Practice in Ghana: Motivating and Demotivating Factors. *Radiol Technol* **2018**, *89*, 337–343.	Full-text not available
4.	Arleo, E. K.; Parikh, J. R.; Wolfman, D.; Gridley, D.; Bender, C.; Bluth, E. Utilization of the Family and Medical Leave Act in Radiology Practices According to the 2016 ACR Commission on Human Resources Workforce Survey. *J Am Coll Radiol* **2016**, *13*, 1440–1446.	Mismatch with the inclusion criteria
5.	Baerlocher, M.O.; Walker, M. Does gender impact upon application rejection rate among Canadian radiology residency applicants?. *Can Assoc Radiol J* **2005**, *56*, 232–237.	Full-text not available
6.	Beeler, W.H.; Griffith, K.A.; Jones, R.D.; Chapman, C.H.; Holliday, E.B.; Lalani, N.; Wilson, E.; Bonner, J.A.; Formenti, S.C.; Hahn, S.M.; et al. Gender, Professional Experiences, and Personal Characteristics of Academic Radiation Oncology Chairs: Data to Inform the Pipeline for the 21st Century. *Int J Radiat Oncol Biol Phys* **2019**, *104*, 979–986.	Mismatch with the inclusion criteria
7.	Belinsky, S.B.; Blagg, J.D. Analyzing the glass ceiling effect among radiologic technologists. *Radiol Technol* **2011**, *82*, 300–310.	Full-text not available
8.	Bendels, M.; Müller, R.; Brueggmann, D.; Groneberg, D.A. Gender disparities in high-quality research revealed by Nature Index journals. *PLoS One* **2018**, *13*, e0189136.	Mismatch with the inclusion criteria
9.	Bluth, E.I.; Cox, J.; Bansal, S.; Green, D. The 2015 ACR Commission on Human Resources Workforce Survey. *J Am Coll Radiol* **2015**, *12*, 1137–1141.	Insufficient information
10.	Boechat M.I. Women in pediatric radiology. *Pediatr Radiol* **2010**, *40*, 484–487.	Mismatch with the inclusion criteria
11.	Bouchardy, J.; Testuz, A.; Blanche, C. Gender aspects in cardiac imaging. *Cardiovasc Med* **2019**, *22*, w02069.	Mismatch with the inclusion criteria
12.	Boulis A. The Evolution of Gender and Motherhood in Contemporary Medicine. Ann Am Acad Pol Soc Sci 2004, 596, 259–261.	Mismatch with the inclusion criteria
**13.**	Branstetter, B. F4th.; Faix, L.E.; Humphrey, A.L.; Schumann, J.B. Preclinical medical student training in radiology: the effect of early exposure. *AJR Am J Roentgenol* **2007**, *188*, W9–W14.	Mismatch with the inclusion criteria
14.	Bundy, B.D.; Bellemann, N.; Weber, M.A. Vereinbarkeit von Familie und ärztlichem Beruf [Compatibility of family and medical profession]. *Radiologe* **2011**, *51*, 801–803.	Mismatch with the inclusion criteria
15.	Campbell, J.C.; Yoon, S.C.; Grimm, L.J. Collaboration Metrics Among Female and Male Researchers: A 5-Year Review of Publications in Major Radiology Journals. *Acad Radiol* **2018**, *25*, 951–954.	Mismatch with the inclusion criteria
16.	Chan, H.F.; Torgler, B. Gender differences in performance of top cited scientists by field and country. *Scientometrics* **2020**, *125*, 2421–2447.	Mismatch with the inclusion criteria

17. Choi, M.; Fuller, C.D.;Thomas, C.R. Estimation of citation-based scholarly activity among radiation oncology faculty at domestic residency-training institutions: 1996-2007. *Int J Radiat Oncol Biol Phys* **2009**, *74*, 172–178.	Mismatch with the inclusion criteria
18. Estrada-Ramírez, K.P.; Miranda-Lora, A.L.; Sandoval-Quiroa, R.C.; Ávila-Montiel, D.; Mier-Prado, M.J.; Garduño-Espinosa, J. Gender gap in the authorship of published articles in the Boletín Médico del Hospital Infantil de México. *Bol Med Hosp Infant Mex* **2018**, *75*, 216–223.	Mismatch with the inclusion criteria
19. Ekpo, E.U.; Snaith, B.; Harris, M.A.; McEntee, M.F. Doctoral profile of the medical radiation sciences: a baseline for Australia and New Zealand. *J Med Radiat Sci* **2017**, *64*, 195–202.	Mismatch with the inclusion criteria
20. Frandsen, T.F.; Jacobsen, R.H.; Ousager, J. Gender gaps in scientific performance: a longitudinal matching study of health sciences researchers. *Scientometrics* **2020**, *124*, 1511–1527.	Mismatch with the inclusion criteria
21. Giner-Soriano, M.; López-Pereiro, O.; Zabaleta-Del-Olmo, E.; Pons-Vigués, M.; Morros, R.; Gómez-Lumbreras, A. Análisis bibliométrico de la autoría femenina en artículos originales en la revista ATENCIÓN PRIMARIA [Bibliometric analysis of female authorship in original articles in the journal ATENCIÓN PRIMARIA]. *Aten Primaria* **2019**, S0212-6567(19), 30423–8.	Mismatch with the inclusion criteria
22. Grimm, L.J.; Ngo, J.; Pisano, E.D.; Yoon, S. Men (and Women) in Academic Radiology: How Can We Reduce the Gender Discrepancy?. *AJR Am J Roentgenol* **2016**, *206*, 678–680.	Mismatch with the inclusion criteria
23. Grimm, L.J.; Redmond, R.A.; Campbell, J. C.; Rosette, A. S. Gender and Racial Bias in Radiology Residency Letters of Recommendation. *J Am Coll Radiol.* **2020**, *17*, 64–71.	Mismatch with the inclusion criteria
24. Grissom, N.M.; Reyes, T.M. Let's call the whole thing off: evaluating gender and sex differences in executive function. *Neuropsychopharmacology* **2019**, *44*, 86–96.	Mismatch with the inclusion criteria
25. Guss, Z.D.; Chen, Q.; Hu, C.; Guss, L.G.; DeWeese, T.L.; Terezakis, S. A. (2019). Differences in Physician Compensation Between Men and Women at United States Public Academic Radiation Oncology Departments. *Int J Radiat Oncol Biol Phys* **2019**, *103*, 314–319.	Mismatch with the inclusion criteria
26. Hamberg K. Gender bias in medicine. *Womens Health (Lond)* **2008**, *4*, 237–243.	Mismatch with the inclusion criteria
27. Hamid, S.; Ali Inam, S.H.; Jamil, H.; Zeb, R. Speciality preference with respect to gender among medical students of Pakistan. *J Pak Med Assoc* **2019**, *69*, 1190–1193.	Mismatch with the inclusion criteria
28. Hamidizadeh, R.; Jalal, S.; Pindiprolu, B.; Tiwana, M.H.; Macura, K.J.; Qamar, S.R.; Nicolaou, S.; Khosa, F. Influences for Gender Disparity in the Radiology Societies in North America. *AJR Am J Roentgenol* **2018**, *211*, 831–838.	Insufficient information
29. Harrington, S.G.; Harvey, H.B. Quality Improvement and Reimbursements: An Opportunity to Address Health Disparities in Radiology. *J Am Coll Radiol* **2019**, *16*, 635–637.	Mismatch with the inclusion criteria
30. Heiligers, P.J.; de Jong, J.D.; Groenewegen, P.P.; Hingstman, L.; Völker, B.; Spreeuwenberg, P. Is networking different with doctors working part-time? Differences in social networks of part-time and full-time doctors. *BMC Health Serv Res* **2008**, *8*, 204.	Mismatch with the inclusion criteria
31. Hinze, S.W. Inside Medical Marriages: The Effect of Gender on Income. *Work and Occupations* **2000**, *27*, 464–99.	Mismatch with the inclusion criteria
32. Holliday, E.B.; Jagsi, R.; Wilson, L.D.; Choi, M.; Thomas, C.R.; Fuller, C.D. Gender differences in publication productivity,	Mismatch with the inclusion criteria

	academic position, career duration, and funding among U.S. academic radiation oncology faculty. *Acad Med* **2014**, *89*, 767–773.	
33.	Ibn Auf, A.; Awadalla, H.; Ahmed, M. E.; Ahmed, M. H. Comparing the participation of men and women in academic medicine in medical colleges in Sudan: A cross-sectional survey. *J Educ Health Promot* **2019**, *8*, 31.	Mismatch with the inclusion criteria
34.	Jalilianhasanpour, R.; Chen, H.; Caffo, B.; Johnson, P.; Beheshtian, E.; Yousem, D.M. Are Women Disadvantaged in Academic Radiology? *Acad Radiol* **2020**, *27*, 1760–1766.	Full-text not available
35.	Jutras, M.; Malekafzali, L.; Jung, S.; Das, P.; Qamar, S.R.; Khosa, F. National Institutes of Health: Gender Differences in Radiology Funding. *Acad Radiol* **2020**, *S1076-6332(20)*, 30482–7.	Full-text not available
36.	Kalaitzi, S.; Czabanowska, K.; Fowler-Davis, S.; Brand, H. Women leadership barriers in healthcare, academia and business. *Equality, Diversity and Inclusion* **2017**, *36*, 457-474.	Mismatch with the inclusion criteria
37.	Madsen, L.B.; Kalantarova, S.; Jindal R.; Akerman, M.; Fefferman, N.R.; Hoffmann, J.C. National survey to assess gender, racial, and ethnic differences among radiology residency applicants regarding factors impacting program selection. *Acad Radiol* (in press).	Full-text not available
38.	Martin, C. A.; Woodring, J. H. Attitudes toward women in radiology. *J Am Med Womens Assoc* **1972**, *41*, 50–53.	Full-text not available
39.	McClelland, S.; Holland, K.J. You, Me, or Her: Leaders' Perceptions of Responsibility for Increasing Gender Diversity in STEM Departments. *Psychology of Women Quarterly* 2014, *39*, 210–225.	Mismatch with the inclusion criteria
40.	Moghimi, S.; Khurshid, K.; Jalal, S.; Qamar, S.R.; Nicolaou, S.; Fatima, K.; Khosa, F. Gender Differences in Leadership Positions Among Academic Nuclear Medicine Specialists in Canada and the United States. *AJR Am J Roentgenol* **2019**, *212*, 146–150.	Mismatch with the inclusion criteria
41.	Morris, E.; Kubik-Huch, R.A.; Abdel-Wahab, M.; Balogun, E.; Beardmore, C.; Beets-Tan, R.; Boyd-Thorpe, A.; Derchi, L.; Fuchsjäger, M.; Husband, J.; Jackson, V.; et al. Women in focus: advice from the front lines on how to enable well-being and build resilience. *Insights Imaging* 2020, *11*, 55.	Mismatch with the inclusion criteria
42.	Obodovskiy, I. *Radiation: Fundamentals, Applications, Risks, and Safety*; Publisher: Elsevier, Netherlands, 2019.	Mismatch with the inclusion criteria
43.	Osborn, V.W.; Doke, K.; Griffith, K.A.; Jones, R.; Lee, A.; Maquilan, G.; Masters, A.H.; Albert, A.A.; Dover, L.L.; Puckett, L.L.; et al. A Survey Study of Female Radiation Oncology Residents' Experiences to Inform Change. *Int J Radiat Oncol Biol Phys* **2019**, *104*, 999–1008.	Mismatch with the inclusion criteria
44.	Perez, Y.V.; Kesselman, A.; Abbey-Mensah, G.; Walsh, J. A Glance at Gender-Specific Preferences Influencing Interventional Radiology Selection. *J Vasc Interv Radiol* **2016**, *27*, 142–143.e1.	Mismatch with the inclusion criteria
45.	Pfleiderer, B.; Bortul, M.; Palmisano, S.; Rodde, S.; Hasebrook, J. Improving female physician's careers in academic medicine: chances and challenges. *Best Pract Res Clin Anaesthesiol* **2018**, *32*, 15–23.	Mismatch with the inclusion criteria
46.	Rodrigues, J.J. *Advancing Medical Practice through Technology: Applications for Healthcare Delivery, Management, and Quality.* Publisher:IGI Global, USA, 2013.	Mismatch with the inclusion criteria

47. Rosen, M.P.; Davis, R.B.; Lesky, L.G. Utilization of outpatient diagnostic imaging. Does the physician's gender play a role?. *J Gen Intern Med* **1997**, *12*, 407–411.	Mismatch with the inclusion criteria
48. Safdar, B.; Greenberg, M.R. Applying the gender lens to emergency care: from bench to bedside. *Acad Emerg Med* **2014**, *21*, 1325–1328.	Mismatch with the inclusion criteria
49. Sepulveda, K.A., Paladin, A.M., Rawson, J.V. Gender Diversity in Academic Radiology Departments: Barriers and Best Practices to Optimizing Inclusion and Developing Women Leaders. *Acad Radiol* **2018**, *25*, 556–560.	Mismatch with the inclusion criteria
50. Spalluto, L.B.; Arleo, E.K.; Lewis, M.C.; Oates, M.E.; Macura, K.J. Addressing Needs of Women Radiologists: Opportunities for Practice Leaders to Facilitate Change. *Radiographics* **2018**, *38*, 1626–1637.	Mismatch with the inclusion criteria
51. Strax, R. Diversity Committee: A Means to Local Gender Equity. *J Am Coll Radiol* **2019**, *16*, 407.	Mismatch with the inclusion criteria
52. Vosper M.R.; Price R.C.; Ashmore L.A. Careers and destinations of radiography students from the University of Hertfordshire. *Radiography* **2005**, *11*, 79-88.	Mismatch with the inclusion criteria
53. Webb, E.M.; Bucknor, M.D.; Naeger, D.M. Diversity and Inclusion: Now Radiology Must Walk the Walk. *J Am Coll Radiol* **2018**, *15*, 687–688.	Mismatch with the inclusion criteria
54. Weigel, K.S.; Kubik-Huch, R.A.; Gebhard, C. Women in radiology: why is the pipeline still leaking and how can we plug it? *Acta Radiol* **2020**, *61*, 743–748.	Mismatch with the inclusion criteria

Supplementary Table S2. Quality appraisal of the included studies

MMAT Tool

Qualitative Studies

	1. Is the qualitative approach appropriate to answer the research question?	2. Are the qualitative data collection methods adequate to address the research question?	3. Are the findings adequately derived from the data?	4. Is the interpretation of results sufficiently substantiated by data?	5. Is there coherence between qualitative data sources, collection analysis, and interpretation?
Piltch-Loeb et al., 2020	✓	✓	✓	✓	✓

Quantitative descriptive studies

	1. Is the sampling strategy relevant to address the research question?	2. Is the sample representative of the target population?	3. Are the measurements appropriate?	4. Is the risk of nonresponse bias low? (for case series and case report: are there complete data on the cases?)	5. Is the statistical analysis appropriate to answer the research question?
Abdellatif et al., 2019	✓	✓	✓	NA	✓
Abduljabbar et al., 2020	✓	✓	✓	✓	✓
Ahmadi et al., 2017	✓	✓	✓	NA	✗
Arleo et al., 2016	✓	✓	✓	✗	✗
Baker et al., 2006	✓	✓	✓	NA	✗
Battaglia et al., 2018	✓	✓	✓	NA	✓
Bernhard et al., 2020	✓	✓	✓	NA	✓
Bluth et al., 2015	✓	✓	✗	✗	✗
Buddeberg-Fischer et al., 2012	✓	✓	✓	✗	✓
Campbell et al., 2017	✓	✓	✓	NA	✓
Campbell et al., 2018	✓	✓	✓	NA	✓
Cater et al., 2018	✓	✓	✓	NA	✓
Chapman et al., 2014	✓	✓	✓	NA	✓
Chertoff et al., 2001	✓	✓	✓	✓	✓
Daldrup-Link et al., 2019	✓	✓	✓	✓	✓
Deipolyi et al., 2020	✓	✓	✓	NA	✓
Deitch et al., 1998	✓	✓	✓	✓	✓
Dial et al., 1989	✓	✓	✓	NA	✗
Donovan 2010	✓	✓	✓	✓	✓
Duc et al., 2020	✓	✓	✓	NA	✓
Fielding et al., 2007	✓	?	✓	✓	✗
Foo et al., 2020	✓	✓	✓	✗	✓
Frank et al., 1999	✓	✓	✓	NA	✗
Grimm et al., 2017	✓	✓	✓	✗	✓
Hewett et al., 2016	✓	✓	✓	NA	✓

Joshi et al., 2020	✓	✓	✓	NA	✓
Kapoor et al., 2016	✓	✓	✓	NA	✓
Kapoor et al., 2017	✓	✓	✓	NA	✓
Kurshid et al., 2018	✓	✓	✓	NA	✓
Lewis et al., 2007	✓	✓	✓	✓	✓
Li et al., 2020	✓	?	?	NA	✗
Liang et al., 2015	✓	✓	✓	NA	✓
Maddu et al., 2020	✓	✓	✓	NA	✓
Magnavita et al., 2013	✓	?	✓	?	✓
McDonald et al., 2017	✓	✓	✓	NA	✓
O'Connor et al., 2018	✓	✓	✓	NA	✓
O'Neill et al., 2019	✓	✓	✓	NA	✓
Owen et al., 1995	✓	✓	✓	✓	✓
Piper et al., 2016	✓	✓	✓	NA	✓
Piper et al., 2018	✓	✓	✓	NA	✓
Pyatigorskaya et al., 2017	✓	✓	✓	✓	✓
Pyatrigorskaya et al., 2017	✓	✓	✓	NA	✓
Qamar et al., 2020	✓	✓	✓	NA	✓
Qamar et al., 2018	✓	✓	✓	NA	✓
Rosenkrantz et al., 2018	✓	✓	✓	NA	✓
Rosenkrantz et al., 2019	✓	✓	✓	NA	✓
Roubidoux et al., 2009	✓	✓	✓	✗	✓
Sadigh et al., 2019	✓	✓	✓	NA	✓
Shah et al., 2007	✓	✓	✓	✗	✓
Vernuccio et al., 2019	✓	✓	✓	?	✓
Vydareny et al., 2000	✓	✓	✓	?	✓
Wah & Belli, 2018	✓	✓	✓	✗	✗
Wang et al., 2018	✓	✓	✓	NA	✓
West & Nguyen, 2016	✓	✓	✓	NA	✓
Whitley et al., 1987	✓	✓	✓	?	✓
Xiao et al., 2018	✓	✓	✓	NA	✓
Yun et al., 2015	✓	✓	✓	NA	✓
Zener et al., 2016	✓	✓	✓	✗	✓
Zulfiqar et al., 2020	✓	✓	✓	NA	✓

Mixed methods studies

	1. Is there an adequate rationale for using a mixed methods design to address the research question?	2. Are the different components of the study effectively integrated to answer the research questions?	3. Are the outputs of the integration of qualitative and quantitative components adequately interpreted?	4. Are divergences and inconsistencies between quantitative and qualitative results adequately addressed?	5. Do the different components of the study adhere to the quality criteria of each tradition of the methods involved?
Englander et al., 2018	✓	✓	✓	✓	✗

✓= Yes; ✗= No; ? = Can’t tell ; NA = not applicable
